# Supplementary material for: A 20-Year Longitudinal Study of Plasma Chitotriosidase Activity in Treated Gaucher Disease Type 1 and 3 Patients—A Qualitative and Quantitative Approach
Source: Biomolecules. 2023 Feb 24;13(3):436. doi: 10.3390/biom13030436 (PMC10046580; doi:10.3390/biom13030436)
Supplement: Supplementary file 1 [file biomolecules-13-00436-s001.zip › biomolecules-2167954-supplementary.pdf]

Table S1. Genotypes of patients with Gaucher disease enrolled into the study.

Genotype	Number of patients
Gaucher disease type 1	
N370S/L444P	23
N370S/G377S	1
N370S/84GG	1
G377S/G377S	1
R433S/R433S	1
R48W/84GG	1
Gaucher disease type 3	
L444P/L444P	19
